# Physical Exercise during Encoding Improves Vocabulary Learning in Young Female Adults: A Neuroendocrinological Study

**DOI:** 10.1371/journal.pone.0064172

**Published:** 2013-05-20

**Authors:** Maren Schmidt-Kassow, Marie Deusser, Christian Thiel, Sascha Otterbein, Christian Montag, Martin Reuter, Winfried Banzer, Jochen Kaiser

**Affiliations:** 1 Institute of Medical Psychology, Goethe University, Frankfurt am Main, Germany; 2 Institute of Sports Sciences, Department of Sports Medicine, Goethe University, Frankfurt am Main, Germany; 3 Institute of Personality and Biological Psychology, University of Bonn, Bonn, Germany; 4 Center for Economics and Neuroscience, University of Bonn, Bonn, Germany; University of California, San Francisco, United States of America

## Abstract

Acute physical activity has been repeatedly shown to improve various cognitive functions. However, there have been no investigations comparing the effects of exercise during verbal encoding versus exercise prior to encoding on long-term memory performance. In this current psychoneuroendocrinological study we aim to test whether light to moderate ergometric bicycling during vocabulary encoding enhances subsequent recall compared to encoding during physical rest and encoding after being physically active. Furthermore, we examined the kinetics of brain-derived neurotrophic factor (BDNF) in serum which has been previously shown to correlate with learning performance. We also controlled for the BDNF val66met polymorphism. We found better vocabulary test performance for subjects that were physically active during the encoding phase compared to sedentary subjects. Post-hoc tests revealed that this effect was particularly present in initially low performers. BDNF in serum and BDNF genotype failed to account for the current result. Our data indicates that light to moderate simultaneous physical activity during encoding, but not prior to encoding, is beneficial for subsequent recall of new items.

## Introduction

There is increasing evidence that single bouts of physical exercise improve cognitive functions. Exercise has been shown to increase the speed of information processing [1]–[3], improve executive function; such as in the Eriksen flanker task, Trail making test, or Stroop interference [4], [5], enhanced cognitive flexibility [6], as well as working memory [7], [8]. However, there is opposing evidence concerning long-term memory. On the one hand, acute exercise has been found to have little or no impact on long-term memory. Winter and colleagues [9] tested young and healthy male recreational athletes with a within-subjects learning paradigm, where participants had to learn new vocabulary either (i) after being sedentary, (ii) after treadmill running at high intensity, or (iii) after treadmill running at moderate intensity. They found that neither high- or moderate-intensity exercise prior to learning led to enhanced vocabulary retention at the same day, after 1 week or after 8 months. Coles and Tomporowski [10] also applied a within-subjects design to investigate the influence of acute exercise on cognitive tasks including a free-recall test. This test was applied prior to and after the following interventions: (i) 40 minutes of continuous cycling at moderate intensity, (ii) 40 minutes of sitting on a cycle ergometer, and (iii) 40 minutes of watching an educational documentary. When comparing performance before and after the exercise intervention, acute exercise did not enhance recall performance. However, in comparison to the other interventions, acute exercise could offset the recall decline observed in the non-exercise conditions.

On the other hand Labban and Etnier [11] provided evidence for a significant exercise-induced memory improvement as tested by a paragraph recall. The authors compared three experimental conditions in a between-subjects design: (i) 30 minutes of cycling at moderate intensity prior to exposure and 30 minutes of rest after exposure (ii) 30 minutes of rest prior and 30 minutes of cycling at moderate intensity after exposure and (iii) 30 minutes of rest prior and after exposure. The acute exercise bout prior to exposure but not after exposure resulted in significantly higher memory performance. Hence the timing of exercise relative to a memory task modulates its effect. Additionally, Salas et al. [12] provide evidence that walking prior to encoding but not prior to retrieval enhances performance in a free recall task. Hence, acute exercise seems to be particularly beneficial if temporally close to encoding. Furthermore, our own study [13] which will be described in more detail, showed that light to moderate intensity exercise during vocabulary encoding increases vocabulary retrieval performance. In search of physiological parameters that mediate the effect of exercise on mnemonic processes, Griffin et al. [14] have recently shown that acute exercise results in a significant increase of brain-derived neurotrophic factor (BDNF) circulating in serum. Serum BDNF was positively correlated with performance in a face-name-matching task known to involve the hippocampus. In a similar vein, Winter et al. [9] found that vocabulary learning was accelerated in participants with higher serum BDNF levels. These results are in line with recent research on the role of BDNF in the regulation of neuroplasticity. A growing number of studies have indicated that BDNF levels are associated with cognitive processes such as memory (e.g., [15], [16]). A high expression of BDNF mRNA has been found both in the cerebral cortex and hippocampus [17], [18]. Along these lines, Hariri et al. [19] have shown that memory-related hippocampal activity during encoding and retrieval correlates positively with the individual peripheral BDNF level. Although the sources of peripheral BDNF are still not fully identified, BDNF is known to bidirectionally cross the blood-brain barrier and thus a substantial part of BDNF in serum may be released centrally (e.g. [20]). Acute exercise bouts have repeatedly been shown to elevate both serum and plasma BDNF in humans, most of them indicating that exercise needs to be at least of moderate intensity (e.g., [21]–[24], [18], [25], [9]). These findings suggest that single exercise bouts represent an attractive tool to boost cognitive functions via enhanced circulating BDNF. However, there are alternative accounts explaining the positive effect of exercise on cognition such as the neurotransmitter hypothesis [26], the arousal hypothesis (e.g. [27]), or the oxygen saturation hypothesis (e.g., [28]).

A number of studies (e.g. [29]–[31]) have shown that a common single nucleotide polymorphism of the BDNF gene (val66met) with a prevalence of 20–30% in the Caucasian population (according to [32]) affects peripheral BDNF levels. BDNF val66met is located on the human chromosome 11p14 leading to an exchange of the amino acid valine to methionine, resulting in decreased BDNF levels for met allele carriers (genotypes met/met or val/met) compared to val/val homozygotes. There is increasing evidence suggesting that the mentioned BDNF polymorphism may play a role in learning and long-term memory ([30], [33], [34] but see also [35], [36] for contradictory findings). This is also supported by findings from genetic imaging studies showing that the met allele is associated with lower gray matter volume in several areas of the temporal lobe including the hippocampus (e.g. [37], [38]).

The current study aims to resolve some of the open questions mentioned above. We assessed how acute exercise and in particular exercise during encoding affects the memorization of new vocabulary, and whether possible effects are related to peripheral BDNF levels and to the BDNF val66 met polymorphism as so far only one study has investigated the influence of acute exercise and peripheral BDNF on verbal learning [9]. Recently we have found that exercising *during* vocabulary learning at a light to moderate intensity level resulted in better performance compared to learning while being physically inactive [13]. In that combined electroencephalographic and behavioral study we observed both higher accuracy in vocabulary tests and larger N400 effects in a cross-language priming paradigm for the group which was asked to exercise on a cycle ergometer during auditory vocabulary learning. However, in this study we neither recorded endocrinological parameters nor did we include a pre-learning exercise group. Hence, our results were not directly comparable to those of previous studies. On the one hand, exercise during encoding may be particularly effective due to the increased elevation of serum BDNF. Recently, we found that serum BDNF rapidly declines within the first 10 minutes of recovery, after exercise [24]. Hence, participants who exercise prior to learning should benefit less from elevated BDNF than those who exercise during learning. On the other hand exercising during encoding provides a dual task situation that may bind attentional resources, resulting in less accurate encoding (as concluded by [33]).

The current study addresses these shortcomings. We investigated whether exercise prior to encoding or exercise during encoding results in better retrieval compared to encoding during physical inactivity. In a young female sample which participated in two learning sessions we investigated how i) simultaneous light to moderate bicycling affects vocabulary learning compared to ii) vocabulary learning after exercise, and iii) learning in a physically relaxed situation. Additionally, we monitored the BDNF level in the second learning session and within a subgroup of participants we determined the individual BDNF genotype. Here, we chose light to moderate exercise intensity although previous studies had provided evidence that exercise needs to be at least of moderate intensity to elevate BDNF in serum. We did so because our previous data had shown that simultaneous exercise at light to moderate intensity enhances long-term memory performance [13]. Given this evidence we hypothesized that light- to moderate-intensity exercise PLUS learning should result in a BDNF elevation comparable to running PLUS an enriched environment as reported in rat studies [39].

We hypothesized that:

1. Participants in the simultaneously bicycling group should maximally benefit from elevated BDNF and hence show increased learning performance compared to the other groups.
2. Subjects in the simultaneously exercising group need to become familiarized with the dual task situation. Therefore, the beneficial effect of simultaneous exercise in contrast to physical activity prior to learning and learning in a physically passive situation should be greater for the second learning session compared to the first learning session, i.e. we expect an interaction between the experimental group and the day of testing.
3. Performance in vocabulary tests should positively correlate with serum BDNF levels during encoding.
4. Homozygotic val/val participants should show higher BDNF levels and thus better performance compared to met allele carriers.

## Materials and Methods

### Participants

105 right-handed (as determined by the Edinburgh handedness inventory [40]) monolingual German young and healthy females (aged between 18 and 30 years; for more details see below) volunteered for the current study. Potential participants were excluded if they had a history of psychiatric and neurological disorders, smoked, were currently on medication (except for contraceptives), had any knowledge of Polish or other Slavic languages, or suffered from cardiovascular diseases.

### Ethics Statement

The study was approved by the Ethics Committee of the Goethe University of Frankfurt Medical Faculty and was conducted in accordance to the principles laid down in the Declaration of Helsinki. All subjects were informed about the aims of the study and gave informed written consent.

### Procedure

Subjects were first screened in a pre-experimental evaluation session. Participants which passed this screening were asked to come to our laboratory for two more learning sessions.

### Pre-experimental Screening

Participants were asked to complete several questionnaires. Their physical activity level was measured with the Freiburg Questionnaire of Physical Activity (FQPA; [41]). Furthermore, they had to indicate the number of foreign languages they have learned and the number of musical instruments they played. We did so as musical expertise and foreign language processing are known to interact [42]–[44]. We controlled for the state of health during the last five years using a short questionnaire where we asked for specific disorders which could potentially result from cardio-vascular diseases such as vertigo, impaired vision, chest pain, tachycardia, or dyspnea. We also checked for other chronic diseases such as diabetes, asthma or epilepsy, respiratory disorders, disorders of the musculoskeletal system, as well as surgeries within the last five years, pregnancy, and acute infections. Additionally, participants were asked to indicate whether they take oral contraceptives (OC women) or not (non-OC women).

Furthermore, we tested the candidates’ ability to memorize new vocabulary. Here, they were asked to listen to 40 pseudowords followed by a German counterpart. All vocabulary pairs were presented via headphones. After thirty minutes during which participants continued to fill in the questionnaires listed above, subjects took part in a vocabulary test, i.e. they were presented the pseudowords and had to write down the associated German words. Candidates who were able to memorize more than twenty pseudowords were excluded from the main experiment to avoid ceiling effects. Twenty-four participants were excluded after the screening session, leaving N = 81 subjects for the experimental sessions. Subjects were matched into groups of 3 and then randomly assigned to one of the three experimental groups (N = 27 per group), i.e. participants were matched according to age and performance in the pseudoword learning task. Experimental groups will be described in the following.

### Session Protocol & Experimental Groups

**Figure 1:**
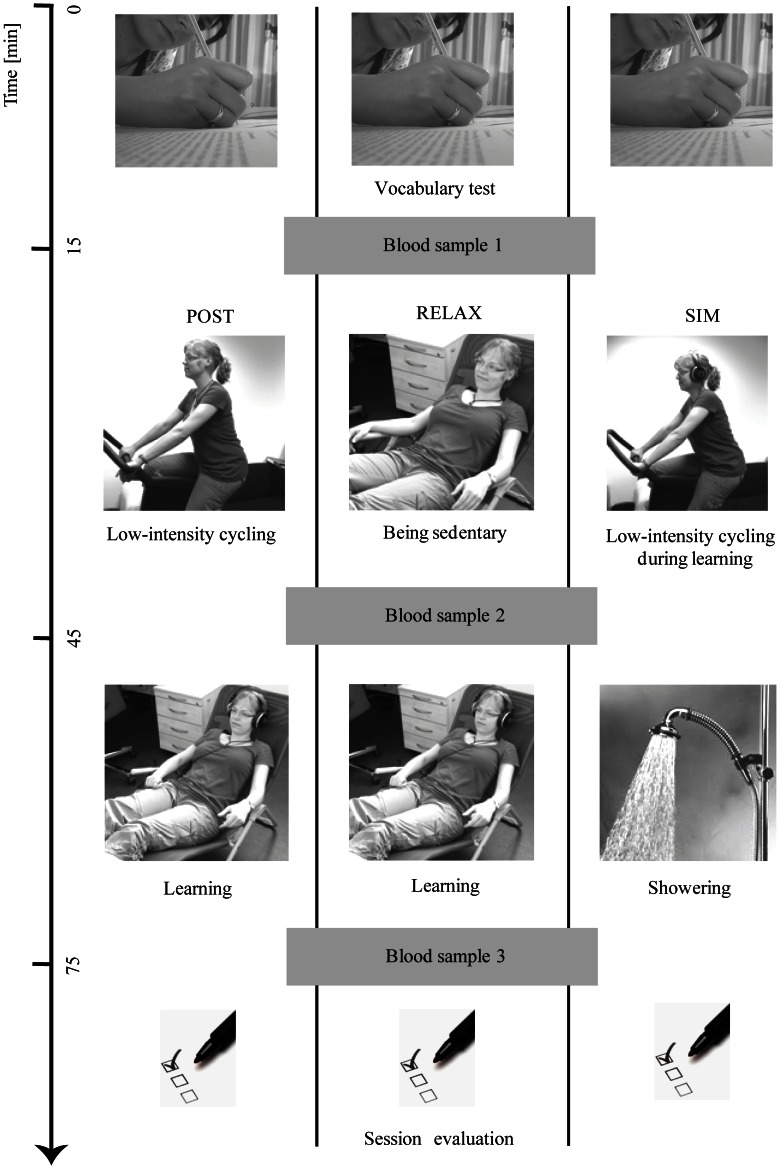
2^nd^ session overview. RELAX = physically inactive group; POST = learning after exercising; SIM = learning during exercising.

We ran two experimental sessions that followed the same learning protocol to control for familiarization effects in the simultaneously exercising group. Blood samples were only taken in the second learning session. We carefully controlled that the second session took place exactly 48 hours after the first. Figure 1 provides an overview of the structure of the second session for each of the groups.

Members of the physically inactive group (“RELAX” group) learned new vocabulary words as described below after resting in a canvas chair for thirty minutes. Participants in the post-exercise group (“POST” group) were asked to exercise on a cycling ergometer (Conditronic 100 PV/ZR-NS, Dynavit, Germany) for thirty minutes before they learned new words, and participants in the simultaneous-exercise group (“SIM” group) exercised on the cycling ergometer while learning the new words. Thirty minutes of exercise intervention have been established as a common protocol for aerobic exercise (see [45] for a recent review) and successfully applied previously ([13]). The rest time between exercise ending and learning onset was no more than 2 min. During this time, a blood sample was taken in the second session (as described below). After pre-experimental screening participants were pseudo-randomly assigned to one of three groups, RELAX, POST, and SIM.

Vocabulary training lasted for 30 minutes. Accordingly, members of the SIM group simultaneously cycled and learned for 30 minutes. Members of the POST group cycled for 30 minutes before they started their vocabulary learning for another 30 minutes. Members of the RELAX group likewise listened to the new vocabulary for thirty minutes after having been sedentary for the same amount of time (Figure 1). After exercising, participants of the SIM group took a shower within a time window of thirty minutes to keep the time frame of blood collection comparable to the other experimental groups. Participants of the POST group were offered a shower after they finished their vocabulary training.

At the end of each session, participants were asked to indicate their *current* level of i) motivation to learn new vocabulary, ii) perceived physical fitness, iii) daily stress, iv) daily workload at university, v) difficulty to learn the new vocabulary, and the vi) quality of their last night’s sleep. Answers were given on a 5-point Likert scale ranging from “low” to “high”. Furthermore, we asked the participants about their caffeine and alcohol consumption, as well as to indicate the number of hours of sleep they received in the previous 24 hours, and to indicate their menstrual phase, i.e. whether they were in the follicular phase, or in the luteal phase, or whether they were currently menstruating.

In the second session, participants completed a paper and pencil vocabulary test as described in the section ‘vocabulary learning’ when they arrived at our lab which took about 12 to 15 minutes. Afterwards, the actual learning session began. Water was provided to all participants throughout the session.

Out of the 81 subjects, 43 agreed to be BDNF-genotyped and gave their written consent. These participants provided buccal cells for genotyping the BDNF val66met polymorphism.

### Exercise Protocol

To regulate exercise intensity in the physically active groups (SIM, POST), we used a 5-point Likert scale (“−2 = extremely light”, “−1 = light”, “0 = moderate”, “+1 = hard”, “+2 = extremely hard”) which has previously been shown to be a reliable and valid tool for measuring perceived exertion [46]. In the initial 5 minutes of each bout, resistance was increased until participants’ perceived exertion reached a light to moderate exertion level. The baseline resistance was 25 Watt. Resistance was increased in 25 Watt steps.

Between consecutive learning runs (see trial overview below) resistance was optionally adjusted to ensure that participants stayed at a light to moderate level of exertion. After completing the whole training session physically active participants were asked to indicate their individual exertion level which did not differ between groups (Median/Mode POST: −1/−1, Median/Mode Sim: −1/−1; p = .9 according to Mann-Whitney test). During the exercise bout, the individuals’ heart rate was constantly monitored (Polar S810, Polar, Büttelborn, Germany). Mean heart rate during exercise was 125 bpm (SD = 26 bpm) for the SIM and 128 bpm (SD = 26 bpm) for the POST group. During learning mean heart rate was 76 bpm (SD: 7 bpm) for the POST group and 74 bpm (SD: 6 bpm) for the RELAX group. Bonferroni-corrected t-tests revealed significant higher heart rates for POST and SIM group compared to RELAX group (both p’s>.001), but no significant effect for the comparison of POST and SIM group (p>.7). Exercise intensity as estimated by a maximum heart rate formula for women exercising on a cycling ergometer [47] revealed an intensity level of 68.9 (SD: 4.09) % HR_max_, which is at the lower edge of moderate intensity according to the guidelines of the ACSM 2011 and hence fits the ratings of perceived exertion. Furthermore, we additionally validated the Likert scale with the Borg RPE scale, which is a more traditional exertion scale, in 30 out of our 87 participants in a graded exercise test. The validation indicates that a rating of “−1” on the used Likert scale corresponds to a value of 12 ( = ″light-moderate”) on a 20-point BORG scale. Together with the estimated %HR_max_ data this result justifies the use of the Likert-scale to assess the participants’ exertion level.

During cycling each revolution was recorded by a customized microcontroller (®Arduino, www.arduino.cc) whenever pedals crossed a light barrier which was built into the cycling ergometer. Due to technical errors, cycling speed and variability had to be excluded in 10 participants from the SIM group and in 11 participants from the POST group. In the remaining subjects’ average cycling speed was 61.04 (SD = 5.9) in the SIM group and 59.55 (5.7) in the POST group, i.e. both groups did not vary significantly (t(31) = 0.75; p = .46).

### Vocabulary Learning

During vocabulary learning, 80 Polish-German vocabulary pairs were presented twice via headphones (AKG K271, HARMAN International Industries, Stamford, USA). The order of vocabulary pairs was randomized for each learning session and each subject. In total, participants heard each vocabulary pair 4 times in total. The loudness level of stimulus presentation was adjusted to the individual preference and kept constant across both learning sessions. Both Polish and German items were spoken by female, non-professional native speakers with a phonetic–linguistic background. All stimuli were normalized to a sound-pressure level of 75 dB using the software PRAAT. The stimulus onset asynchrony (SOA) of Polish–German vocabulary pairs was aligned to the perceptual centre of a word [48], i.e. the vowel onset of the stressed syllable, and amounted to 2 seconds. The SOA between successive vocabulary pairs was 6 seconds. We excluded action verbs to ensure that better performance of the SIM group was not semantically induced [49]. Participants in both physically active groups were offered to cycle at a speed of about 60 revolutions per minute (RPM), a pace that is usually recommended to beginners in fitness centers. To familiarize participants of the SIM group to cycling and listening to auditory stimuli at the same time, we presented sinusoidal tones at a rate of 1 Hz for 2 minutes and at 0.5 Hz for 3 minutes before the actual vocabulary presentation was started. After one run of vocabulary presentation, the same tones were presented for 3 minutes to give participants a break before all vocabulary pairs were presented again in a second run. Forty-eight hours after each learning session subjects participated in a vocabulary test. Here, they listened to all of the Polish vocabulary via headphones and were asked to write down the German translation in a paper form. Response time was limited to 8 s for each item, i.e. after 8 s the next vocabulary was presented. The first vocabulary test (paper and pencil) took place at the beginning of the second learning session. Half of the participants finished the second vocabulary test at our laboratory, the other half completed it online from home for the sake of feasibility. For the online test the presentation of vocabulary was identical, i.e. participants were instructed to use head phones and inter-stimulus-interval was set to 8 s. It was impossible to replay either single words or the whole vocabulary list. The single difference was that participants had to type their answers instead of writing it down.

### Blood Sampling

In the second sessions three venous blood samples were taken from each participant. The first blood sample was collected after the participants completed the first vocabulary test. Hence, cardio-respiratory parameters were on a stable baseline level at the beginning of the training period. The second blood sample was taken after the particular intervention (cycling in the POST group, cycling & learning in the SIM group, or lying in a canvas chair in the RELAX group). The third blood sample was taken 30 minutes after the second (i.e. after the vocabulary learning in the POST and RELAX groups, and after showering in the SIM group).

### Analysis of BDNF Serum Concentrations

In each of the three blood samples, 4.5 ml of venous blood from the antecubital vein was collected with a clotted blood tube. Collection and analysis of blood samples was performed according to the following uniform protocol: all samples clotted within 30 minutes at a temperature of 21C. After the clotting period, samples were centrifuged for 10 minutes with 4800 rounds per minute using the Heraeus Labofuge 200 (Thermo Fisher Scientific, Germany). Immediately afterwards, serum was pipetted into separate SafeSeal micro tubes (Sarstedt, Nürnberg, Germany). Samples were stored at −30 C for one night and then transferred to a −80C freezer for another three weeks. BDNF levels in serum were measured using the Quantikine® Human BDNF Immunoassay from R&D Systems (Wiesbaden, Germany). Serum samples were diluted 1∶20 with Calibrator Diluent. 100 µl Assay Diluent was incubated with a 50 µl standard or diluted sample, respectively, for 2 hours at room temperature. Afterwards, 100 µl of mouse monoclonal antibody against BDNF was conjugated to horseradish peroxidase and incubated for another 1 hour. After washing the plates three times with wash buffer, 200 µl of substrate solution (color reagents A and B mixed in equal volumes) was added and the plates were incubated for 30 min and protected from light. The color reaction was stopped by adding 50 µl of stop solution and the optical density of each well was measured at 450 nm with a microplate reader (TECAN SpectraFluor Plus) within 30 min. Wavelength correction was performed at 540 nm. The minimum detectable BDNF dose was less than 20 pg/mL, according to the manufacturer’s information. All samples were tested twice for reliability.

### Genotyping

DNA was extracted from buccal cells. Automated purification of genomic DNA was conducted by means of the MagNA Pure® LC system using a commercial extraction kit (MagNA Pure LC DNA isolation kit; Roche Diagnostics, Mannheim, Germany). Genotyping of the BDNF val66met polymorphism was performed by real-time polymerase chain reaction (RT-PCR) using fluorescence melting curve detection analysis by means of the Light Cycler System 1.5 (Roche Diagnostics, Mannheim, Germany). The primers and hybridisation probes (TIB MOLBIOL, Berlin, Germany) and the PCR protocol for BDNF val66met are as follows:

forward primer: 5′-ACTCTGGAGAGCGTGAATGG-3′;

reverse primer: 5′-CCAAAGGCACTTGACTACTGA-3′;

anchor hybridisation probe: 5′-LC640-CGAACACATGATAGAAGAGCTGTT-phosphate-3′;

sensor hybridisation probe: 5′-AAGAGGCTTGACATCATTGGCTGACACT-fluorescein-3′.

The PCR run comprised 50 cycles of denaturation (95 C, 0 s, ramp rate 20 C s^−1^), annealing (55 C, 10 s, ramp rate 20 C s^−1^), acquisition of the fluorescence signal (55 C, 1 s, ramp rate 20 C s^−1^) and extension (72 C, 12 s, ramp rate 20 C s^−1^), which followed an incubation period of 10 min (90 C) to activate the FastStart Taq DNA Polymerase of the reaction mix (Light Cycler FastStart DNA Master Hybridization Probes, Roche Diagnostics, Mannheim, Gemany). After amplification, a melting curve was generated by keeping the reaction time at 40 C for 2 min and then heated slowly to 75 C with a ramp rate of 0.2 C s^−1^. The fluorescence signal was plotted against temperature to yield the respective melting points (*T*_m_) of the two alleles. *T*_m_ was 58.5 C for the val allele and 63.8 C for the met allele.

### Data Analysis

Performance in the vocabulary tests was compared between experimental groups using a 2*3 repeated measures ANCOVA with the factors day of testing (day1, day2) and experimental group (POST, SIM, RELAX) as independent variables and the factors contraception (OC, non-OC) and menstrual phase (follicular, luteal, menorrhea) as covariates. We included contraception and menstrual phase as covariates as previous studies showed steroid hormones and menstrual phase to affect learning performance and neural substrates relevant for learning (e.g. [50], [51]).

To investigate the influence of BDNF on learning performance, we calculated the logarithm of mean BDNF (average of time point 1, 2, and 3), BDNF at each time point separately (1, 2, 3), BDNF kinetics for learning (BDNF after learning minus BDNF at baseline) and BDNF kinetics for exercise (BDNF after exercise minus BDNF at baseline) for each participant and computed correlations with vocabulary test performance for each experimental group. As previous studies demonstrated an effect of menstrual phase and oral contraceptives on BDNF levels (e.g. [52] we computed a 3*3 ANCOVA on logBDNF with the within-subject factor time point (1,2,3), the between-subject factor experimental group (POST, RELAX, SIM) and the covariates menstrual phase (follicular, luteal, menorrhea) and contraception (OC, non-OC).

We grouped met/met and val/met genotypes to increase statistical power. Hence, we computed a one-way ANOVA with the dependent variable log mean BDNF and the independent variable genotype (val/val homozygotes versus met allele carriers). We also computed a separate 2*2 repeated measures ANOVA with the factors day of testing (day1, day2) and genotype (val/val homozygotes versus met allele carriers) to evaluate the influence of BDNF genotype on BDNF in serum levels and on learning performance.

To test the effect of individual’s innate ability to learn vocabulary on the relationship between exercise and learning, we performed a post-hoc median split analysis on performance in the pre-experimental pseudoword learning paradigm as described in the section on *pre-experimental screening.* Participants with fewer than ten memorized words were then labeled as “low performers” and participants with more than ten memorized words were labeled as “high performers”. Sibley and Beilock [8] have shown that the beneficial effect of an acute bout on the treadmill on working memory performance, measured by the reading span and the operation span, was restricted to those participants with the lowest working memory capacities (for a review see [53]). Referring to Sibley and Beilock, we conducted a 2*2*3 ANCOVA with the factors day of testing (day1, day2), performance on the pseudoword learning test (high, low) and experimental group (POST, SIM, RELAX) with the covariates menstrual phase (follicular, luteal, menorrhea) and contraception (OC, non-OC).

## Results

### Participants

The subjects’ age ranged from 19 to 29 years (Mean POST: 22.7 years, SD = 2.1, Mean RELAX = 22.8, SD = 2.0, Mean SIM = 23.4, SD = 3.1 ), 51 were OC women, 30 were non-OC women (POST: 19 OC/8 non-OC; RELAX: 17 OC/10 non-OC; SIM: 15 OC/12 non-OC), their mean BMI was below 22 (Mean POST: 21.8, SD = 1.4, Mean RELAX = 21.2, SD = 1.7, Mean SIM = 21.2, SD = 1.6 ), and their FQPA level indicated that they were moderately physically active (mean: 49 metabolic equivalent-hours per week (MET-h/wk); SD: 31.6, POST: 51.5 MET-h/wk (34.7), SIM: 42.1 MET-h/wk (22.9), RELAX: 53.6 MET-h/wk (35.4). On average, subjects were familiar with 3 foreign languages (Median POST: 3; Median SIM: 3; Median RELAX: 3) and played 2 musical instruments (Median POST: 1; Median SIM: 2; Median RELAX: 2).

The POST, SIM and RELAX groups did not differ in their ability to memorize vocabulary (pseudoword test, p>.1), age (p>.5), BMI (p>.2), or FQPA (p>.3), number of foreign languages (p>.1), or number of musical instruments played (p>.2).

Concerning the session questionnaires there were no group differences in their current level of perceived physical fitness (Median of all groups: high, p>.1), daily stress (Median POST: low; Median SIM: low; Median RELAX: neither high nor low, p>.3), daily workload at university (Median POST: rather high; Median SIM: neither high nor low; Median RELAX: low, p>.1), difficulty to learn the new vocabulary (Median of all groups: neither high or low, p>.1), quality of last night’s sleep (Median POST: good; Median SIM: good; Median RELAX: rather good; p>.06); all p>.06). Furthermore participants did not differ in caffeine (Median POST: 1cup, Median SIM: 0.5 cup, Median RELAX: 0.5 cup; p>.3) or alcohol consumption (Median all groups: 0 glasses; p>.1 ), nor did they differ in the number of hours slept within the last 24 hours (Mean all groups: 7 h (SD: 0.8); p>.3).

During the experimental sessions, 34 participants indicate to be in the follicular phase (POST: 11; RELAX: 13; SIM: 10), while 40 participants were in the luteal phase (POST: 15; RELAX: 11; SIM: 14), and 7 were menstruating (POST: 1; RELAX: 3; SIM: 3).

### Performance

The omnibus ANCOVA revealed a main effect of day of testing (F(1,76) = 37.2, p<.001, η_p_^2^ = .329; mean day 1: 6.89 (SD: 3.96) words; mean day 2: 25.35 (SD: 10.34) words) and an interaction of day of testing × experimental group (F(2,76) = 3.87, p = .02, η_p_^2^ = .092; see Table 1).

**Table 1:** Vocabulary test performance - whole group analysis: Means, standard deviations and p-values for significant experimental effects.

Source	Condition	p	Mean (SD)
day of testing		<.001	
	day 1		6.89 (3.96)
	day 2		25.35 (10.34)
day of testing*experimental group		.02	
experimentalgroup-day 1		.06	
	SIM vs RELAX	<.05	8.2 (4.3); 5.6 (3.0)
	POST vs RELAX	n.s.	6.8 (4.1); 5.6 (3.0)
	SIM vs POST	n.s.	8.2 (4.3); 6.8 (4.1)
experimentalgroup-day 2		.02	
	SIM vs RELAX	<.02	28.4 (9.8); 20.9 (7.9)
	POST vs RELAX	n.s.	26.6 (11.7); 20.9 (7.9)
	SIM vs POST	n.s.	28.4 (9.8); 26.6 (11.7)

**Figure 2:**
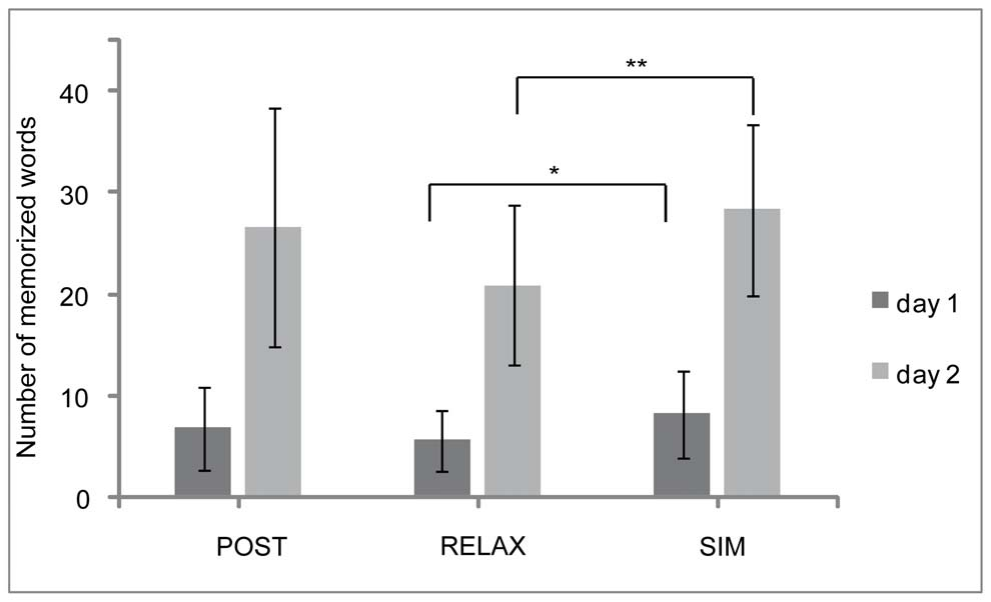
Vocabulary test performance for each day and experimental group. Error bars indicate standard deviations.

Resolving the interaction revealed a trend for experimental group for the first day and significant effect for experimental group on the second day (day 1: F(2,76) = 2.9, p = .06, η_p_^2^ = .07; day 2: F(2,76) = 4.23, p = .02, η_p_^2^ = .1; see Figure 2). Bonferroni-corrected post-hoc tests revealed better performance in both vocabulary tests for the SIM group (mean day 1 = 8.2 (SD = 4.3) words; mean day 2 = 28.4 (SD = 9.8) words) compared to the RELAX group (mean day 1 = 5.6 (SD = 3.0) words; t(52) = 2.593, d = 0.71 p<.05; mean day 2 = 20.9 (SD = 7.9) words; t(52) = 3.065, d = 0.84, p<.02). Bonferroni-corrected contrasts on difference (test2 minus test1) revealed a larger difference between each day of testing for the SIM group compared to the RELAX group (t(52) = 2.75, d = 0.76, p<.05).

Performance of the POST group (mean day 1 = 6.8 (SD = 4.1); mean day 2 = 26.6 (SD = 11.7)) did not differ from either the RELAX or the SIM group (p>.05).

Additionally, to test whether performance in the second vocabulary test was influenced by the test setting (online versus in the laboratory) we ran an ANOVA with the between-subject factors group (SIM, POST, RELAX) and setting (online, laboratory) on vocabulary test performance. This analysis revealed no significant main effect of setting nor an interaction between group and setting (p = .2).

### Serum BDNF & Genotyping

Mean BDNF in serum values ranged between 13412.2 and 56169.5 pg/ml (mean: 31627.7 pg/ml SD: 8486.8).

LogBDNF in serum did not differed between experimental groups at any time point (p = .37, see Table 2) nor was there a positive correlation between vocabulary test performance in any group and BDNF in serum at any time (p>.3) or BDNF kinetics (p>.2), except for BDNF kinetics during exercise. Here, we found a significant increase for the POST group compared to the RELAX group (t(52) = 4.86; d = 1.3, p<.01) and a marginally significant change for the SIM group compared to the RELAX group (t(52) = 2.43; d = 0.67, p = .02). Exercise groups did not vary in BDNF change in response to exercise (p>.05). Bonferroni-corrected alpha was. 016.

**Table 2:** Mean logBDNF in serum as well as standard deviations for each experimental group and time point.

Group	Time point	logBDNF
RELAX		
	1	10.32 (.29)
	2	10.25 (.26)
	3	10.30 (.27)
POST		
	1	10.19 (.33)
	2	10.37 (.42)
	3	10.25 (.33)
SIM		
	1	10.38 (.25)
	2	10.43 (.24)
	3	10.37 (.28)

The genotype frequencies were in Hardy–Weinberg equilibrium: val66val: *n* = 26, met allele carriers (val66met & met66met): n = 17; Chi^2^ = 2.61, df = 1, n.s.

Log mean BDNF in serum was lower (t(41) = 1.704, d = 0.53, p = .05, one-tailed) for met-allele carriers (mean = 10.19, SD = 0.33, N = 17) than for val homozygotes (mean = 10.37, SD = 0.29, N = 26).

### Performance and Genotyping

Performance in the vocabulary test did not vary as a function of BDNF genotype (F(1,1) = .004; p = .9).

### Pseudoword Learning Performance

Overall, low and high performers differed significantly in their test performance (test 1: low performers: 4.6 (3); high performers: 9.1 (3.5); t(79) =  = 6.1; p<.001; test2: low performers: 17.8 (7); high performers: 32.6 (7.3); t(79) = 9.3; p<.001).

**Figure 3:**
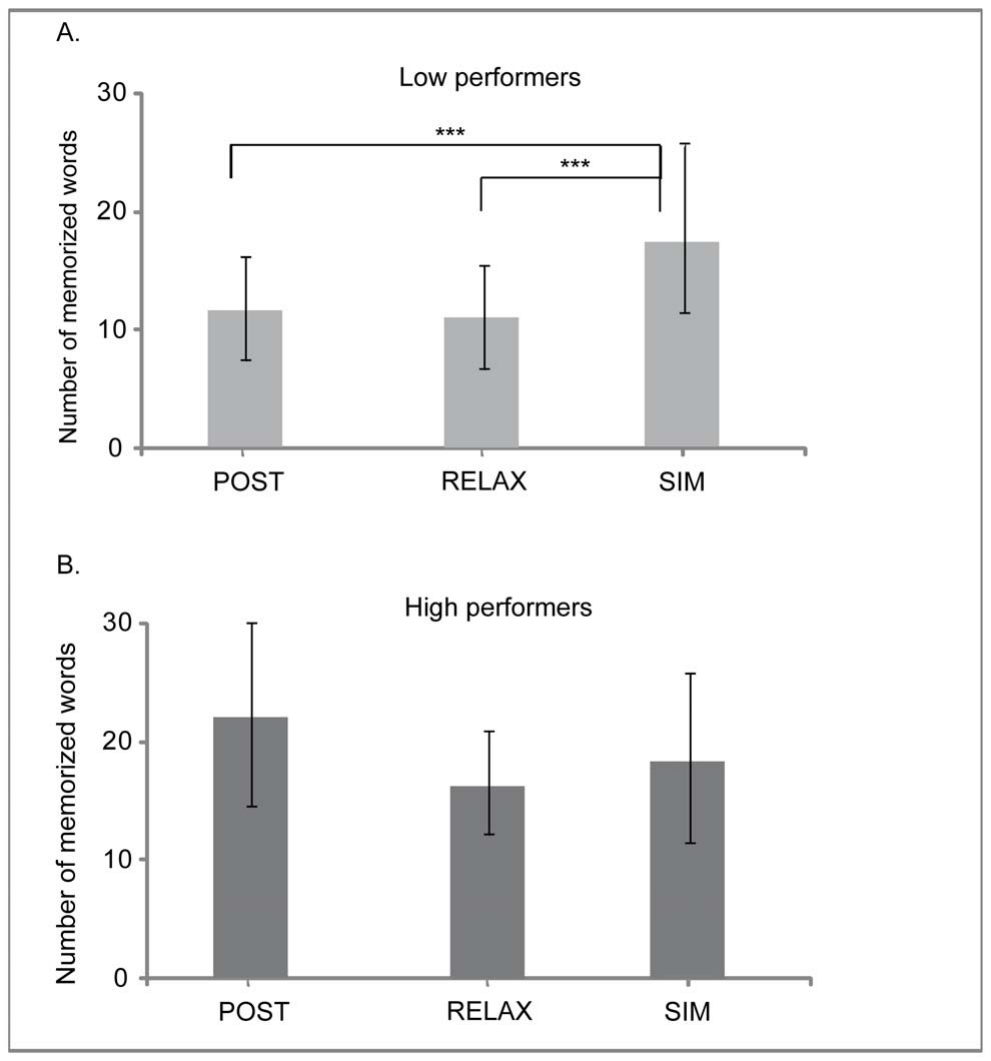
Vocabulary test performance averaged across the two days of testing for each experimental group for low (panel A) and high performers (panel B) separately. Error bars indicate standard deviations.

The exploratory 2x2x3 ANCOVA with the factors day of testing, pseudoword learning performance, and experimental group resulted in a significant interaction between experimental group and pseudoword learning performance (F(2,73) = 3.9, p = .02, η_p_^2^ = .097; see Table 3). Bonferroni-corrected post-hoc tests on the performance averaged across the two days of testing revealed significantly better performance for the SIM group (N = 10; mean = 17.8 (1.6) words) compared to the other learning conditions (RELAX: N = 17; mean 11.3 (1.2) words; t(25) = 3.0, d = 1.2, p<.01; POST: N = 14; 12 (1.4) words; t(25) = 2.6, d = 1.04, p = .01) for the “low performer” group, but no differences between experimental groups for high performers (p = .2; SIM: N = 17; mean = 18.6 (1.6) words, RELAX: N = 10; mean = 16.7 (2.1) words; POST: N = 13; mean = 21.8 (1.8) words; see Figure 3).

**Table 3:** Vocabulary test performance concerning pseudoword learning performance: Means, standard deviations and p-values for significant experimental effects.

Source	Condition	p	Mean (SD)
experimental group × pseudoword learning performance		.02	
experimental group-low performers		.01	
	SIM vs RELAX	.01	17.8 (1.6); 11.3 (1.2)
	POST vs RELAX	n.s.	12 (1.4); 11.3 (1.2)
	SIM vs POST	.01	17.8 (1.6); 12 (1.4)
experimental group-high performers		n.s.	
	SIM vs RELAX	n.s.	18.6 (1.6); 16.7 (2.1)
	POST vs RELAX	n.s.	21.8 (1.8); 16.7 (2.1)
	SIM vs POST	n.s.	18.6 (1.6); 21.8 (1.8)

## Discussion

The current experiment was designed (i) to compare the effect of exercise prior to encoding and exercise during encoding with being sedentary during encoding on vocabulary retrieval. (ii) To replicate the positive correlation of peripheral BDNF and vocabulary learning performance, and (iii) to assess the effects of the BDNF val66met polymorphism. We found that light- to moderate-intensity physical activity during encoding improved vocabulary learning in young, healthy women. As hypothesized, this effect is larger after the second learning session, indicating that subjects of the simultaneously exercising group benefited from a familiarization phase. We did not find significant correlations between serum BDNF and learning performance. Although this is in contrast to the results from other studies demonstrating a clear positive correlation between cognitive functions and peripheral BDNF (e.g. [14], [19], [9]), the present findings are in line with our previous observations that only highly intense physical activity led to transient BDNF elevations, which disappeared after exercise [24]. Possibly the present exercise was not intense enough to produce a clear effect of BDNF on performance.

On the other hand, it is conceivable that word retrieval during the vocabulary test has influenced baseline BDNF in serum differently between the experimental groups. Particularly for the SIM group, word retrieval might have increased BDNF in serum as participants were physically active during vocabulary encoding. Recently, Macedonia et al [54] found retrieval-related activity in the premotor cortices for words that were previously encoded with simultaneous body gestures. Hence, motor involvement during encoding can involve motor activation in a physically passive retrieval situation, which in turn might result in elevated BDNF level. Nonetheless, we feel confident that this feature of our study has not influenced or even abolished BDNF effects for the following reason: if the vocabulary test had an impact on serum BDNF, BDNF at baseline should have differed between experimental groups, i.e. there should have been higher baseline values for SIM compared to the other conditions. However, we found no group differences at any time point. Hence, even if vocabulary retrieval has influenced BDNF in serum this should apply equally to all of the experimental groups.

We found that BDNF genotype was unrelated to learning performance. Thus, we are confident that even if homozygotic participants had been over-represented in the SIM group this could not explain their better performance. On a critical note, the number of genotyped subjects was rather small and the lack of a group difference might be due to insufficient statistical power.

The current results confirm our previous findings that female participants benefit from simultaneous low-intensity ergometer exercise during vocabulary encoding [13]. Interestingly, we obtained comparable effect sizes as provided by Labban & Etnier [11]. Hence less intense exercise may also affect memory performance resulting in medium to large positive effect, depending on the occurrence of exercise intervention (d = 0.7–0.8; [55]). Also in line with the aforementioned authors, we failed to find differences between both exercise conditions (SIM vs POST). This might be due to individual differences in learning preferences. Many participants anecdotically reported that they like to move during learning while others strictly refuse any kind of distraction during learning. However, the mechanism that mediates these differences is still unknown. Possibly, salivary cortisol as a physiological marker of individual stress [56] might be suitable to shed light on this issue. Salivary cortisol may reveal the participants’ stress level in response to a simultaneous exercise and learning task [57]. Indeed Almela et al. [58] have shown that a higher cortisol response to the stressor negatively influences memory performance in middle-aged women. However, others have provided evidence for stress-enhanced memory effects (e.g. [59]). A follow-up within-subject experiment to investigate this issue is currently being conducted.

An exploratory analysis as to the possible effects of general vocabulary learning ability revealed that for participants with a lower verbal memory capacity, simultaneous exercise leads to superior performance compared to both exercise prior to encoding and resting. Apparently, simultaneous exercise enables low performers to increase their performance to a level comparable to high performers. These results support data from Sibley and Beilock [8] showing that exercise is particularly beneficial for subjects with low working memory skills. Similarly, for carriers of the apolipoprotein E ε4 allele (a known risk factor for Alzheimer’s disease) regular physical activity has been demonstrated to be advantageous for efficient stimulus processing in a working memory task [60]. The authors provided magnetoencephalographic evidence that carriers of the ε4 allele which are highly physically active show similar processing speeds to non-carriers while ε4 allele carriers with low physical activity were significantly slower in comparison to the non-carrier counterparts. In summary, physical activity might be particularly beneficial for subjects with lower memory, and less effective for subjects with higher memory capacities.

In the current study we examined whether BDNF is responsible for the positive effect of exercise on cognition supporting neuronal growth and differentiation. We failed to find a clear involvement of BDNF in the relationship of exercise and cognition. As we did not collect other measures of neuroendocrines, conclusions about why simultaneous exercise improved verbal learning have to remain speculative. An increased level of catecholamines may play a role in this context [61], however, this needs to be addressed in future research. At least two other hypotheses have been proposed to explain the positive effect of simultaneous exercise on verbal learning. First, increased arousal during exercise may improve cognition. In a review, Tomporowski [62] reported that a number of studies demonstrated that the relationship between cognition and physical arousal follows the Yerkes-Dodson law [63]. Levitt & Gutin [64] reported faster reaction times for moderately increased heart rates, but slower reaction times at highly increased heart rates. This is in line with a model provided by Kahneman [27] claiming that cognitive resources available for task performance are dependent on the participant’s arousal level while moderate arousal levels should result in optimal performance. Our data can also be explained in terms of arousal. Apparently, simultaneous exercise positively affects learning performance, particularly in low performers. If exercise is interpreted as a potential stressor increasing arousal, simultaneous light- to moderate-intensity exercise should increase the arousal level which in turn increases the resources available to perform a cognitive task. If on the other hand exercise withdraws resources necessary to perform the cognitive task, this should result in interference. In this case, the mental workload required by exercise is too high to manage the cognitive task (cf. [65]). Evidently, simultaneous bicycling did not interfere with verbal encoding in our paradigm. While one might argue that the missing interference effect is specific to our female sample, in a recent, as yet unpublished study we found no effects of sex on performance. Hence, in terms of an arousal model the low workload of ergometric cycling may have prevented an interference effect but increased cognitive resources available for encoding. Although exercise intensity might not have been on an optimally high level, the current intensity level should nevertheless have resulted in higher arousal compared to the physically relaxed situation. Hence, following the arousal hypothesis learning should be enhanced due to increased arousal even at a light to moderate intensity level, although the benefit might have been even higher at a more intense level.

Second, the rhythmic stimulus presentation in the current experiment may represent an alternative factor underlying the beneficial effect of simultaneous exercise on verbal learning. As described in the methods section, stimuli were presented every 2 and 6 seconds, respectively. Hence, as participants cycled at a frequency of approximately 1 Hz, they moved in synchrony to the stimulus presentation. Previous work [66]–[69] has provided clear electrophysiological evidence for a processing benefit for temporally predictable compared to temporally unpredictable acoustic stimuli. This was not only true for simple tones, but also for acoustically more complex stimuli, i.e. syllable sequences [70]. The observed effects support the account of a dynamic allocation of attention, i.e. attention should be shifted to prospective points in time when relevant events are expected to occur. This should lead to an increased allocation of cognitive resources for stimulus processing resulting in the facilitated detection of target stimuli. In terms of this attention allocation model, better performance of the SIM group due to rhythmic stimulus presentation would result from the possibility of auditory-motor synchronization. Data from the present experiment can neither support nor refute the arousal hypothesis or the attention allocation hypothesis. Here, follow-up studies are necessary to disentangle the effects of arousal, enhanced BDNF level, and rhythmic stimulation.

On a critical note, one limitation of the current study is that exercise intensity was not determined on the basis of quantitative measures of workload but on the basis of perceived exertion. However, previous studies [71], [72] have reported that ratings of perceived exertion are a reliable tool to assess exercise intensity. The lack of a statistical difference in heart rate, physical activity level, and change in BDNF between the exercise groups support the validity of our procedures.

Another open question concerns the neural substrates of the reported behavioral evidence as none of the reported studies have applied any imaging methods. However, fMRI results from Breitenstein et al. [73] have shown that the retrieval of new vocabulary relies on hippocampal activity during encoding. In good learners, hippocampal activity was enhanced at the beginning of the encoding session, and decreased as learning progressed, while hippocampal activity of poor learners remained on a stable but lower level. Hence, vocabulary learning should be sensitive to interventions that promote hippocampal activity such as physical activity [74]–[78]. Interestingly there is evidence that hippocampal activity is modulated by arousal in a U-shaped manner (see [79] for a review). With regards to the present effects in low performers, simultaneous exercise may have affected hippocampal activity during encoding which in turn may have compensated for these participants’ memory deficit. However, the discussed differences between high and low performers are based on a first explorative analysis. Follow-up studies are necessary which systematically evaluate these differences by carefully preselecting participants with high and low memory skills. More challenging though is investigating of the effect of physical activity on hippocampal activity, as a paradigm like the one presented here is not feasible as an fMRI experiment.

### Conclusions

In summary, in line with previous findings [13] our results support a positive effect of light to moderate exercise while encoding new vocabulary. The related measures BDNF in serum and BDNF genotype failed to account for the observed variance in vocabulary test performance. Post-hoc comparisons between high and low performers revealed a beneficial effect of simultaneous exercise while learning compared to both exercise prior to learning and resting, for the low performers only. Further research is need to decipher the mechanisms modulating this effect.
